# Macronutrient Intake from Human Milk, Infant Growth, and Body Composition at Term Equivalent Age: A Longitudinal Study of Hospitalized Very Preterm Infants

**DOI:** 10.3390/nu12082249

**Published:** 2020-07-28

**Authors:** Mandy Belfort, Sara Cherkerzian, Katherine Bell, Betina Soldateli, Erika Cordova Ramos, Caroline Palmer, Tina Steele, Hunter Pepin, Deirdre Ellard, Kaitlin Drouin, Terrie Inder

**Affiliations:** 1 Department of Pediatric Newborn Medicine, Brigham and Women’s Hospital, Boston, MA 02115, USA; scherkerzian@bwh.harvard.edu (S.C.); kbell7@partners.org (K.B.); betinasoldateli@hotmail.com (B.S.); palmer.caro@gmail.com (C.P.); tdufresne@bwh.harvard.edu (T.S.); hpepin1@bwh.harvard.edu (H.P.); dellard1@bwh.harvard.edu (D.E.); kdrouin@bwh.harvard.edu (K.D.); tinder@bwh.harvard.edu (T.I.); 2 Harvard Medical School, Boston, MA 02115, USA; 3 Division of Newborn Medicine, Boston Children’s Hospital, Boston, MA 02115, USA; Erika.CordovaRamos@childrens.harvard.edu; 4 Department of Nursing, Brigham and Women’s Hospital, Boston, MA 02115, USA; 5 Department of Nutrition, Brigham and Women’s Hospital, Boston, MA 02115, USA

**Keywords:** human milk, infant growth, macronutrient

## Abstract

The variable macronutrient content of human milk may contribute to growth deficits among preterm infants in the neonatal intensive care unit (NICU). In a longitudinal study of 37 infants < 32 weeks gestation, we aimed to (1) determine the between-infant variation in macronutrient intake from human milk and (2) examine associations of macronutrient intake with growth outcomes. We analyzed 1626 human milk samples (median, 43 samples/infant) with mid infrared spectroscopy. Outcomes at term equivalent age were weight, length, head circumference, fat mass, and fat-free mass. Median (range) intakes from human milk were: protein 1.37 (0.88, 2.43) g/kg/day; fat 4.20 (3.19, 5.82) g/kg/day; carbohydrate 8.94 (7.72, 9.85) g/kg/day; and energy 82.5 (68.7, 99.3) kcal/kg/day. In median regression models adjusted for birth size and gestational age, and other covariates, greater intakes of fat and energy were associated with higher weight (0.61 z-scores per g/kg/day fat, 95% CI 0.21, 1.01; 0.69 z-scores per 10 kcal/kg/day, 95% CI 0.28, 1.10), whereas greater protein intake was associated with greater body length (0.84 z-scores per g/kg/day protein, 95% CI 0.09, 1.58). Higher fat intake was also associated with higher fat mass and fat-free mass. Macronutrient intakes from human milk were highly variable and associated with growth outcomes despite routine fortification.

## 1. Introduction

Over 63,000 very preterm (<32 weeks gestation) infants are born each year in the United States [1]. These infants are uniquely vulnerable to undernutrition during the 2–4 month-long neonatal intensive care unit (NICU) hospitalization, which coincides with a critical period for the development of multiple organ systems [2,3,4]. Human milk is the recommended NICU diet based on strong evidence for short- and long-term benefits, such as a reduced risk for life-threatening intestinal complications and better neurodevelopmental outcomes [5,6]. Because human milk alone is insufficient to support optimal postnatal growth and tissue accretion at the same rate as the in-utero fetus [7,8,9], the addition of multicomponent fortifiers to human milk is standard in clinical practice [10]. These fortifiers are designed to meet recommended nutrient targets when added to milk with average nutrient values from published references [7,11] fed at typical daily volumes.

Although NICU growth outcomes have improved in the past two decades [4,12], we and others have documented slower weight gain and head growth among fortified human milk-fed infants as compared with preterm formula-fed infants [3,13,14,15,16], suggesting that current fortification strategies may not meet nutrient requirements for all infants. This observation is particularly important given the substantial rise in the use of both maternal and pasteurized donor human milk (“donor milk”) in NICUs in the past decade [17].

Ensuring adequate macronutrient delivery for human milk-fed preterm infants is challenging. NICU clinicians rely on published references [7,11] to calculate daily nutrient intakes but the macronutrient content of human milk is in fact highly variable. For example, one large study [18] of 736 maternal milk samples reported a 5-fold range in protein and a 10-fold range in fat content. Donor milk is also variable and has less protein on average than maternal milk [19,20]. Milk content is variable whether produced by a mother who delivered preterm or full term, but the impact of the variability is greater for preterm infants who are tube fed while in the NICU and unable to regulate their intake volume to meet nutrient and energy demands. It is therefore possible that some human milk-fed infants develop cumulative deficits in protein and/or energy intake that contribute to impaired growth.

To inform more effective fortification strategies, we need to understand how the day-to-day variation in human milk composition translates to variability in cumulative macronutrient *intakes* between infants over the months-long NICU hospitalization. Currently, knowledge is limited due to a lack of studies with longitudinal data on actual human milk macronutrient content, the ingested volume of human milk, and relevant growth outcomes. We also need to understand the extent to which differences in macronutrient intakes from milk drive growth outcomes in an era of routine fortification.

Recently, the availability of point-of-care human milk analyzers has made it possible to address these knowledge gaps [21]. We conducted a longitudinal study of hospitalized human milk-fed preterm infants with the following aims: (1) to determine the variation in day-to-day and average macronutrient and energy intakes from human milk; and (2) to examine the associations of average macronutrient and energy intakes from human milk with infant size and body composition outcomes at term equivalent age.

## 2. Materials and Method

### 2.1. Population

We conducted a prospective, longitudinal study of infants born <32 weeks gestation in a single academic Level III NICU from 2015 to 2018. We approached parents of 192 eligible infants and parents of 103 consented for their infant(s) to participate. Reasons for not participating included no interest in research, concern about milk supply, plans to transfer to another hospital, and not wanting additional tests for the infant. Of the 103 participants initially enrolled, we excluded 2 who died, 4 who were subsequently diagnosed with a congenital anomaly (an exclusion criteria), and 4 who were transferred to another hospital shortly after enrollment. Of the remaining 93, to facilitate precise estimation of macronutrient intakes from human milk over time, for this analysis, we included 37 infants who received maternal milk only (no formula or donor milk supplementation), had 21 or more milk samples collected and analyzed during their NICU stay, and completed at least one outcome assessment (anthropometry and/or body composition. As compared with the original cohort, the participants included in this analysis had similar mean gestational age (28.2 vs. 28.5 weeks) and birth weight for gestational age z-score (−0.08 vs. −0.14). During the study period, parenteral nutrition, enteral volume advancement, and multicomponent bovine-based liquid human milk fortifier use were in keeping with a local written clinical practice guideline, available upon request. The clinical guideline also covers the use of modular macronutrient fortifiers such as liquid protein and medium chain triglyceride oil, which are used in the case of fluid volume restriction and/or growth faltering.

This study protocol was approved by the Partners Human Research Committee and parents of participating infants provided written informed consent.

### 2.2. Milk Collection and Analysis

As soon as possible after delivery, mothers received standard written and verbal instructions by a board-certified lactation consultant to express milk at least 8 times per day with a double-electric breast pump, augmented with hand expression to improve emptying. We started sampling unfortified milk for research once mothers were producing enough to meet their infants’ needs. One time per day, bedside nurses collected daily 3–5 mL samples of hand-warmed and gently mixed maternal milk prior to fortifying it. This milk may have been pooled from more than one pumping session, typically contained foremilk and hind milk, and may have included fresh and/or previously frozen milk. This pragmatic approach was intended to yield milk samples that represented the infants’ actual diets. All milk was maternal milk, not donor milk. Milk samples collected at the bedside were refrigerated at 4 °C until analysis for macronutrient content by a research assistant, which was performed each weekday.

We used a mid-infrared spectroscopy-based human milk analyzer (Miris AB, Uppsala, Sweden). This point-of-care device estimates crude protein from total nitrogen content using a conversion factor of 6.38 [22], and then calculates the “true” protein as crude protein*0.8 to exclude non-protein nitrogen. Energy (kcals) is calculated by the human milk analyzer from macronutrient values using the following formula: (9.25*fat + 4.40*crude protein + 4.00*carbohydrate [23]. In keeping with manufacturer instructions [23], we sonicated 3 mL of milk for 5 s and then warmed it to 40 °C using a customized Penguin hospital-grade milk warmer (Ameda, Buffalo Grove, IL, USA) prior to analysis. We analyzed each sample of unfortified milk once. Performance testing of our analyzer in collaboration with the MAMAS study [24] revealed high accuracy (r = 0.99 true protein vs. elemental analysis, r = 0.98 fat vs. Mojonnier [25] and reliability (coefficient of variation 5.2% protein, 1.6% fat; email communication with Ms. Celia Kwan, February 2017). From 37 participating infants, we included nutrient data from 1626 milk samples. We excluded extreme nutrient values (>3 standard deviations from the mean) that likely represented measurement errors.

### 2.3. Anthropometry

Infants were weighed at birth and daily to the nearest 1 g on calibrated digital scales (Scale-Tronix, Inc, White Plains, NY, USA) by bedside nurses who recorded weights in the electronic medical record. As close as possible to birth and again at term equivalent age, two authors who are registered dietitians (D.E. and H.P.) measured participants’ body length to the nearest 0.1 cm with a recumbent length board using the 2-person method and head circumference at the largest frontal occipital plane to the nearest 0.1 cm with a non-stretchable tape. For all anthropometric measures, we calculated z-scores for postmenstrual age (PMA) using the Fenton reference [26].

### 2.4. Body Composition

As close as possible to term equivalent age (mean postmenstrual age, 39 completed weeks; range 34 to 42 weeks), we assessed infant body composition with air displacement plethysmography in the Peapod Infant Body Composition system (COSMED USA, Concord, CA, USA). This device uses whole body densitometry and a two-compartment model to estimate fat mass and fat-free mass for infants weighing 1 to 8 kg. The method is safe, feasible, and accurate in the NICU setting [27,28]. The PEA POD was zeroed with any equipment (e.g., feeding tube and oxygen cannula) that could not be removed during the body composition assessment. We calculated z-scores for postmenstrual age using log-transformed values for fat mass and fat-free mass plus 10 based on recently published reference data [29].

### 2.5. Covariates

From the electronic medical record, we abstracted the milk volume fed each day and receipt of parenteral nutrition, as well as demographic and health data including gestational age at birth [30], multiple gestation, and infant sex. We also recorded the documented presence of co-morbidities (necrotizing enterocolitis with Bell Stage 2 or higher, intraventricular hemorrhage Grade 3 or 4, culture-proven late onset sepsis, treatment for retinopathy of prematurity) based on standard definitions [31] and the receipt of postnatal steroids, a marker for chronic lung disease. 

### 2.6. Statistical Analysis

For each day that milk was analyzed, we calculated the daily nutrient intake from milk by multiplying the nutrient content times the milk volume divided by the body weight. For example, if a 1 kg infant ingested 130 mL of milk with protein content 1 g/dL, the nutrient intake on that day was (1 g/dL*130 mL)/1 kg = 1.3 g/kg/day. These variables represented the nutrient intakes from human milk but not from fortifiers. The main exposure variables were the median intakes of true protein, fat, carbohydrate, and energy. The main outcomes were weight, length, head circumference, fat mass, and fat-free mass, expressed as raw values and z-scores. We created histograms to visualize the overall distribution of milk content and box and whiskers plots to visualize the extent of within-and between-infant variation in milk nutrient intakes. We also calculated Pearson correlations to quantify the extent to which daily nutrient intakes were related to milk intake volume and nutrient content. Given non-normality of residual distributions for some models, we used median regression to estimate associations of nutrient intakes with median anthropometry and body composition outcomes. We created separate models for each nutrient exposure and adjusted all models for sex, gestational age, and z-score for birth size (weight, length, or head circumference, corresponding to the outcome in that model; body composition models were adjusted using birth weight z-score) and for intrafamilial correlation among siblings due to the large number of twins. We also adjusted for days of parenteral nutrition, using a cube root transformation of this variable due to its highly skewed distribution. We could not also adjust for the time to achieve full enteral feeding because this variable was highly correlated—and therefore collinear—with the days of parenteral nutrition (Spearman r = 0.75). Statistical analyses were performed using Stata software (StataCorp. 2019. Stata Statistical Software: Release 16. College Station, TX: StataCorp LLC) and module QREG2.

## 3. Results

Participant characteristics are shown in Table 1. Mothers were a mean of 34 years old (standard deviation [SD] 5) and 46% were primiparous. Infants were a mean gestational age of 28.2 weeks (range, 23.6, 31.9), with a mean birth weight of 1104 g (range, 410, 2065 g). Just over half (57%) were male. Each infant contributed a median of 43 milk samples (range, 22 to 84) to the analysis. Samples were collected starting at a median of 12 postnatal days of age (range, 5 to 46) and ending at 68 days (range, 39 to 112).

**Figure 1:**
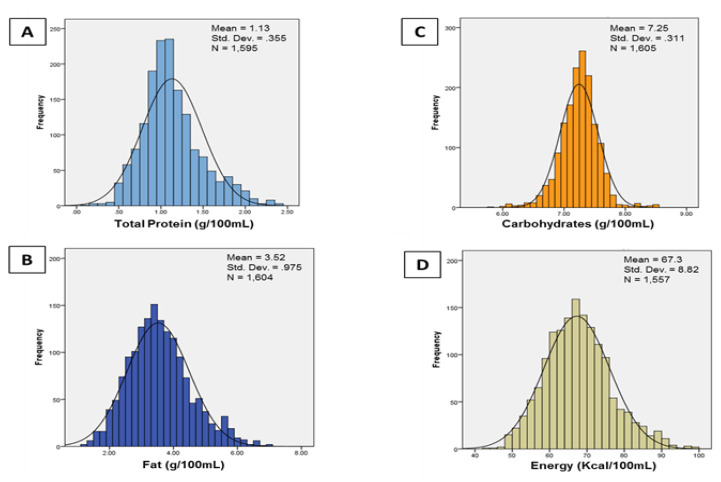
Distributions of protein (panel **A**), fat (panel **B**), carbohydrate (panel **C**), and energy (panel **D**) content in 1626 human milk samples from 37 very preterm infants. Mean (SD) protein content was 1.13 (0.36) g/dL, fat 3.52 (0.98) g/dL, carbohydrate 7.25 (0.31) g/dL, and energy 67.3 (8.8 kcal/dL) or 20.2 (2.6) kcal/oz. Interquartile ranges were: protein 0.90, 1.30 g/dL; fat 2.90, 4.10 g/dL; carbohydrate 7.10, 7.40 g/dL; and energy 61.0, 72.0 kcal/dL. Nutrient content was measured with a mid-infrared spectroscopy-based point of care human milk analyzer (Miris AB, Uppsala, Sweden).

Figure 1 shows the overall distributions of *nutrient content* for 1626 unfortified milk samples. Mean (SD) protein content was 1.13 (0.36) g/dL, fat 3.52 (0.98) g/dL, carbohydrate 7.25 (0.31) g/dL, and energy 67.3 (8.8 kcal/dL) or 20.2 (2.6) kcal/oz. We also calculated the mean nutrient content for each participant (within-participant mean for *n* = 37); the mean for protein content was 1.17 (range, 0.67 to 1.88 g/dL), fat 3.50 (range, 2.60 to 4.63) g/dL, carbohydrate 7.25 (range, 6.69 to 7.68) g/dL and energy 67.3 (range, 57.8 to 79.5) kcal/dL or 20.2 (range, 17.3 to 23.9) kcal/oz. Regarding daily milk intake volume, the within-participant mean intake of unfortified milk ranged from 117 to 133 mL/kg/day (mean, 127 mL/kg/day).

**Figure 2:**
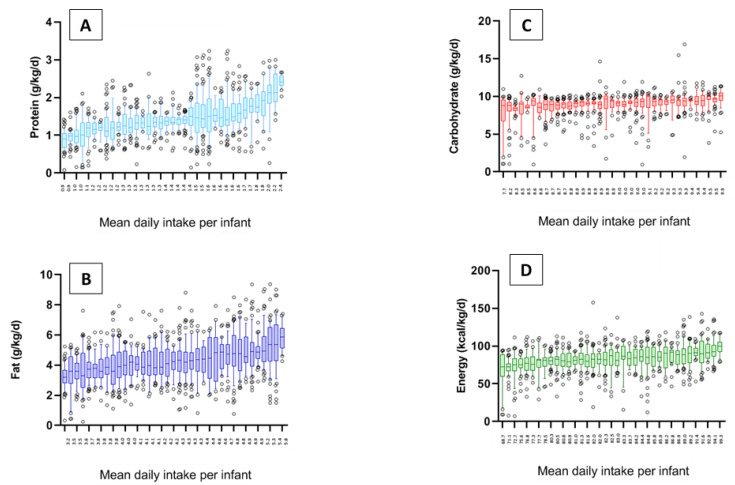
Boxplots showing variation in intakes of protein (panel **A**), fat (panel **B**), carbohydrate (panel **C**), and energy (panel **D**) from unfortified milk. Each boxplot represents one participant (*n* = 37). Day-to-day intakes varied for all participants. Boxplots were ordered by mean daily intake along the x-axis. When taken together, these plots illustrate substantial between-infant variation in average intakes over the NICU hospitalization (range in mean protein intake, 0.9 to 2.4 g/kg per day; fat 3.2 to 5.8 g/kg per day; carbohydrate 7.7 to 9.9 g/kg per day; energy 69 to 99 kcal/kg per day).

Figure 2 illustrates the variation in nutrient intakes from unfortified milk over time. Each box plot displays the within-infant (day-to-day) variation in intake as well as the average intake over all days for that infant. When taken together, the boxplots show substantial between-infant variation in average intake over the study period. Median (range) intakes from human milk were: protein 1.37 (0.88, 2.43) g/kg/day; fat 4.20 (3.19, 5.82) g/kg/day; carbohydrate 8.94 (7.72, 9.85) g/kg/day; and energy 82.5 (68.7, 99.3) kcal/kg/day.

Mean daily nutrient intakes from unfortified milk were strongly correlated with the corresponding mean milk nutrient content. For example, the correlation of mean daily protein intake with mean milk protein content was 0.98 (*p* < 0.0001); and for fat, this correlation was 0.95 (*p* < 0.0001). Mean daily nutrient intakes were only weakly correlated with daily intake volumes. For example, the correlation of mean daily protein intake with mean intake volume (mL/kg/day) was 0.12 (*p* = 0.5); and for fat, this correlation was 0.22 (*p* = 0.18).

Table 2 shows outcome measures including anthropometry and body composition at term equivalent age. All z-scores (except for fat mass z-score) were negative, indicating that, on average, infants in our study were smaller at term equivalent age than the reference population of full-term infants.

Table 3 and Table 4 show the adjusted associations of nutrient intakes with anthropometry and body composition outcomes at term equivalent age. Greater fat and energy intakes were associated with higher weight at term equivalent age (0.62 weight z-scores per g/kg/day fat, 95% CI 0.30, 0.94; 0.62 weight z-scores per 10 kcal/kg/day, 95% CI 0.22, 1.02). Greater protein intake was associated with higher body length (0.99 length z-scores per g/kg/day protein, 95% CI 0.0.10, 1.87). Higher fat intake was also associated with higher fat-free mass (0.57 fat-free mass z-scores, 95% CI 0.37, 0.76), and we noted a similar relationship between energy intake and fat-free mass z-score. Analyses with milk nutrient content (g/dL or kcal/dL) as the exposure variable rather than nutrient intake (g/kg/day) yielded nearly identical results.

## 4. Discussion

Fortified human milk is the recommended diet for hospitalized very preterm infants [5,6] but recent studies [3,13,14,15,16] have reported diminished growth in human milk-fed infants as compared with formula-fed preterm infants, raising concerns regarding the adequacy of current fortification strategies in the NICU. A challenge in clinical practice is the reliance on published reference values for human milk macronutrient content [7,11]. Although numerous studies including a meta-analysis [20] have reported wide variation in the nutrient content of human milk, we investigated between-infant differences in longitudinal nutrient intakes from unfortified milk. Specifically, we leveraged point-of-care milk analyzer technology to investigate the extent to which day-to-day (within-infant) variation in human milk macronutrient content translates to clinically important differences in intake over time.

Across our cohort, we found a nearly 3-fold range in average protein intake (0.88 to 2.43 g/kg) and an approximately 50% range in average energy intake (68.7 to 99.3 kcal/kg) from pre-fortified milk. Importantly, higher mean daily macronutrient and energy intakes were associated with higher body weight and length at discharge despite the routine use of multicomponent human fortifiers, whereas higher fat intake was associated with higher fat mass and fat-free mass. A previous longitudinal study [32] with repeated measures of milk macronutrient content in Taiwan found that the protein content (g/dL) of human milk was positively associated with growth velocity to hospital discharge, a finding that is consistent with our results. Our study substantially extends those results by examining protein and energy intakes measured in g/kg/day, and by assessing linear growth, head growth, and body composition outcomes in addition to weight gain. Taken together, these data suggest that the differences in macronutrient intake from pre-fortified milk are clinically important, even in a setting in which milk fortification is practiced routinely and according to a standardized guideline.

Protein and energy in the diet are major determinants of physical growth for preterm infants [33]. Multicomponent human milk fortification is a routine practice in the United States [10] and is intended to provide the excess nutrients required to support a ‘fetal’ pattern of growth for the 2–4 month period between birth and term equivalent age [7]. In calculating daily nutrient intakes, clinicians rely on published reference values. For example, the American Academy of Pediatrics lists representative values for human milk energy content as 65 to 70 kcal/dL (19.5 to 21 kcal/oz) and protein as 1.6 g/dL in early milk and 0.9 g/dL in mature milk [7]. Instead of relying on these representative values, we directly measured macronutrient content in unfortified milk. Our results demonstrate that when actual daily nutrient contents are averaged over weeks or months for in the NICU, the actual values deviate substantially from representative values in some infants. This deviation is important because clinicians who rely on representative content values rather than actual content values will estimate nutrient intakes incorrectly. For example, an infant receiving unfortified milk with the lowest average protein content observed in our study (0.67 g/dL) would receive 3.8 g/kg/day after standard fortification when fed at 150 mL/kg/day, a protein intake that is well below the recommended intake of 4 to 4.5 g/kg/day [7,11].

In addition to describing the between-infant variation in average actual nutrient content from milk—with relevance to challenges in meeting target nutrient intakes—we found important links between actual nutrient intakes and growth outcomes. Specifically, higher fat and energy intakes predicted greater body weight at discharge and higher protein intake predicted greater body length. Additionally, higher fat intake predicted higher fat-free mass. These findings are generally consistent with studies by McLeod and colleagues [34,35], who reported associations of energy and fat intake with weight gain and fat mass, and of protein intake with weight gain and fat-free mass. Together, these findings support the concept that inadequate protein and energy intakes limit physical growth among some human milk-fed preterm infants, despite routine fortification. These results are particularly significant given the well-established links of slow weight gain and linear growth stunting in the NICU with adverse neurodevelopmental outcomes later in childhood [36,37].

Our results demonstrate that intake differences between infants were driven primarily by variable nutrient content of the milk rather than by variable intake volume. These findings therefore support the concept that intervening with targeted human milk fortification of protein and energy may be an effective strategy to prevent nutrient deficits and improve physical growth, as previously proposed [38]. Thus far only a few clinical trials have examined the effectiveness of this approach [39,40,41,42,43]; results have been inconsistent regarding growth and data are lacking regarding brain development or neurodevelopmental outcomes.

Strengths of our study include its longitudinal design including repeated measurement of macronutrients in milk over the hospitalization and data on actual milk intake volume from the medical record. The combination of data on milk composition and ingested volume enabled us to calculate actual nutrient intakes, rather than relying on published reference values. Most of the milk fed to infants in our study was maternal milk and our findings may not generalize to infants fed predominantly donor milk, which typically has lower protein and energy contents [44]. We included infants fed human milk only, whereas infants fed a mixed diet or formula only may have less variation in nutrient intakes given the consistency in the composition of infant formula. Although clinical measures of infant body length are subject to measurement error, we used a recumbent length board and standardized procedures to minimize this error. We also used state-of-the art methods to estimate body composition including fat mass and fat-free mass. Our sample size was relatively small, which may have limited our ability to detect some true associations. Like all observational studies, ours is subject to residual confounding, and the number of variables we could adjust for was limited due to our sample size. Additionally, we did not have data on potentially confounding factors such as maternal obesity and smoking status, nor did we examine the impact of maternal diet on milk composition.

## 5. Conclusions

In this longitudinal study of hospitalized human milk-fed very preterm infants, we found substantial variation in intakes of protein and energy from human milk. Further, differences in nutrient intake from milk predicted slower weight gain and linear growth, despite routine human milk fortification. These findings suggest that inadequate macronutrient fortification may contribute to poor weight gain and linear growth stunting in some human milk-fed preterm infants and provide support for the investigation of individually targeted human milk fortification as a strategy to reduce macronutrient deficits and improve physical growth in the NICU.

## Figures and Tables

**Table 1:** Participant characteristics (*n* = 37).

**Mothers**			
	*Mean*	*SD*	
Age, years	34	5	
	*Number*	*Percent*	
Race			
White	22	60%	
Black	10	27%	
Asian	2	5%	
Other or missing	3	8%	
Hispanic ethnicity	2	5%	
Primiparous	17	46%	
Antenatal steroids given	36	97%	
Delivery mode			
Vaginal	16	43%	
Cesarean	21	57%	
**Infants**			
	*Mean*	*SD*	*Range*
Gestational age, weeks	28	2.4	23.6, 31.9
Birth weight, grams	1104	434	410, 2065
Birth z-scores			
Weight	−0.08	1.02	−2.36, 1.83
Length	0.03	1.20	−4.00, 1.87
Head circumference	−0.37	0.95	−2.26, 1.87
	*Number*	*Percent*	
Small for gestational age	5	14%	
Sex			
Male	21	57%	
Female	16	43%	
Gestation number			
Singleton	28		
Twins	9		
NEC, Stage 2 or higher	0	0%	
IVH, Grade 3 or 4	1	3%	
Culture proven sepsis	2	5%	
Retinopathy treatment	1	3%	
Postnatal steroids	5	14%	

SD is standard deviation. Birth size z-scores are based on the Fenton reference. Small for gestational age is defined by birth weight < 10th percentile for gestational age, and also based on the Fenton reference. NEC is necrotizing enterocolitis. IVH is intraventricular hemorrhage.

**Table 2:** Anthropometry and body composition outcomes at term equivalent age (*n* = 37).

	*Mean*	*SD*
Raw measurements		
Weight, kg	2.86	0.55
Length, cm	47.2	2.8
Head circumference, cm	33.4	1.6
Fat mass, kg	0.58	0.20
Fat-free mass, kg	2.38	0.40
Percent body fat	19.4	4.8
Z-scores		
Weight	−0.80	1.30
Length	−0.95	1.43
Head circumference	−0.36	1.23
Fat mass	1.99	1.23
Fat-free mass	−1.31	1.52
Percent body fat	2.5	1.2
	*Mean*	*Range*
PMA at outcome, weeks	38.11	34, 42

SD is standard deviation. Z-scores for weight, length, and head circumference based on Fenton [26]. Z-scores for fat mass and fat-free mass based on Norris [32]. PMA is postmenstrual age.

**Table 3:** Associations of nutrient intakes with anthropometry at term equivalent age (*n* = 37).

	Weight z-Score		Length z-Score		Head Circumference z-Score	
	Beta	95% CI	Beta	95% CI	Beta	95% CI
Protein, g/kg/day	0.65	−1.15, 2.44	0.84	0.09, 1.58	−0.05	−0.72, 0.62
Fat, g/kg/day	0.61	0.21, 1.01	0.34	−0.20, 0.88	0.12	−0.31, 0.55
Carbohydrate, g/kg/day	−0.41	−1.63, 0.80	−0.39	−1.09, 0.31	−0.02	−0.67, 0.63
Energy, 10 kcal/kg/day	0.69	0.28, 1.10	0.41	−0.08, 0.90	0.13	−0.28, 0.55

Betas indicate z-score differences in median size at term equivalent age per increment in nutrient intake from unfortified human milk over the study period. Median regression estimates adjusted for gestational age, birth size z-score, sex, and intrafamilial correlation (twins). Bold-face text indicates estimates with confidence intervals that exclude the null hypothesis.

**Table 4:** Associations of nutrient intakes with body composition at term equivalent age (*n* = 30).

	Fat Mass z-Score		Fat-Free Mass z-Score	
	Beta	95% CI	Beta	95% CI
Protein, g/kg/day	0.72	−2.19, 3.63	0.09	−1.29, 1.46
Fat, g/kg/day	0.82	0.13, 1.51	0.54	0.25, 0.83
Carbohydrate, g/kg/day	0.04	−2.32, 2.40	−0.95	−2.04, 0.13
Energy, 10 kcal/kg/day	0.93	−0.05, 1.92	0.49	−0.002, 0.98

Betas indicate z-score differences in median size at term equivalent age per increment in nutrient intake from unfortified human milk over the study period. Median regression estimates adjusted for gestational age, birth size z-score, sex, and intrafamilial correlation (twins). Bold-face text indicates estimates with confidence intervals that exclude the null hypothesis.
